# Immunotherapy in Patients with Advanced Non-Small-Cell Lung Cancer Under-Represented by Clinical Trials

**DOI:** 10.3390/curroncol31090407

**Published:** 2024-09-14

**Authors:** Daniel E. Meyers, Rebekah Rittberg, David E. Dawe, Shantanu Banerji

**Affiliations:** CancerCare Manitoba, Winnipeg, MB R3E 0V9, Canada

**Keywords:** non-small-cell lung cancer, immunotherapy, real-world evidence, clinical trials

## Abstract

Since the initial US FDA approval of an immune checkpoint inhibitor (ICI) for the treatment of non-oncogene-driven non-small-cell lung cancer (NSCLC) nine years ago, this therapeutic strategy has been cemented as a crucial component of treatment for most of these patients. However, there is a clear efficacy–effectiveness gap whereby patients in the ‘real world’ seem to have more modest clinical outcomes compared to those enrolled in landmark clinical trials. This gap may be driven by the under-representation of important patient populations, including populations defined by clinical or molecular characteristics. In this review, we summarize the data outlining the evidence of ICIs in patients with poor Eastern Cooperative Oncology Group performance status (ECOG PS), underlying autoimmune disease (AID), older age, active brain metastases (BMs), and molecular aberrations such as *EGFR* mutations, *ALK* fusions, *BRAF* mutations and *ROS1* fusions.

## 1. Introduction

It has been nine years since an immune checkpoint inhibitor (ICI) first received U.S. Food and Drug Administration (FDA) approval for use in non-oncogene-driven advanced non-small-cell lung cancer (NSCLC). Since that time, numerous phase III trials have established the superior efficacy of these agents compared to cytotoxic chemotherapy in both front- and latter-line settings (Table 1 and Table 2) [1,2,3,4,5,6,7,8,9,10,11,12,13,14,15,16,17,18,19,20,21,22,23]. Several landmark trials have since reported 5-year overall survival (OS) data, which demonstrate how ICIs have revolutionized the treatment paradigm for this disease, with 5-year OS of 18% in KEYNOTE-407 [24], 19% in KEYNOTE-189 [25], 24% in CheckMate 227 [26], and 32% in KEYNOTE-024 [27]. These outcomes are in stark contrast to the reported median 5-year OS of 5.5% in the United States in the pre-ICI era [28].

Despite the clear benefit of ICIs in patients with advanced non-oncogene-driven NSCLC eligible for clinical trials, accumulated evidence from the ‘real world’ suggests a sizeable effectiveness–efficacy gap, with more modest outcomes compared to landmark trials [29,30,31,32,33]. This gap can reasonably be explained by the strict enrollment criteria of many clinical trials, resulting in the exclusion of patients who otherwise make up a substantial proportion of patients seen in everyday clinical practice—such as those with poor Eastern Cooperative Oncology Group performance status (ECOG PS), those with active brain metastases (BMs), and those with autoimmune disease (AID). Indirectly, these and other exclusion criteria lead to the under-representation of older adults [34]. 

While avoiding ICIs in the initial treatment approach to patients with advanced NSCLC and an oncogenic driver—such as an activating *EGFR* mutation [35] or *ALK* fusions [36]—is established, it is still worth considering how these patients fare when treated with ICIs after progression on tyrosine kinase inhibitors (TKIs). Until recently the majority of the data reporting the efficacy of ICIs in oncogene-driven NSCLC have been retrospective, but four recent phase III trials provided important findings with discrepant results [37,38,39,40].

In this review we explore the effectiveness of ICIs in patients who are typically under-represented in landmark clinical trials based on clinical (older age, poor ECOG PS, concomitant AID, or BMs) or molecular factors (*EGFR* mutations, *ALK* fusions, and other oncogenic driving events such as *ROS1* fusions and *BRAF* mutations).

## 2. Clinical Characteristics

### 2.1. Patients with Poor ECOG PS

As the majority of NSCLC is diagnosed at advanced stages, it is unsurprising that ~30% of patients seen in clinical practice have poor ECOG PS (i.e., ECOG PS ≥ 2) at baseline [41]. Nevertheless, the overwhelming majority of phase 3 trials evaluating systemic therapies in NSCLC exclude patients with poor ECOG PS, which has not changed in the ICI era. Importantly, several retrospective studies have identified poor ECOG PS as a strong independent prognostic factor of worse OS in patients with NSCLC treated with ICIs, with reported median OS ranging from 3.0 to 7.4 months [29,30,42,43,44,45]. As such, prospective data to guide decisions around the use of ICIs in patients with advanced NSCLC are desperately needed.

Until recently, the prospective data available in this space were non-randomized and of modest sample size [31,46,47,48,49]. For example, the single-arm PePS2 study enrolled 60 patients with advanced NSCLC and ECOG PS of 2. They reported a mOS of 9.8 months (95% CI 7.1–14.6) in their cohort, with heterogeneity in the line in which the ICI was received and tumor PD-L1 expression [47]. In addition, CheckMate 153 [31] (phase 3b/4) and CheckMate 171 [46] (phase 2) enrolled patients with advanced NSCLC treated with nivolumab in the second (or later) line. Reported mOS for the patients with ECOG PS of 2 in these trials were 4.0 months (95% CI 3.1–6.2) and 5.2 months (95% CI 3.0–7.6), respectively. Most recently, Shaverdashvili et al. published results from their single-arm phase 2 trial evaluating the outcomes of first-line durvalumab in patients who had an ECOG PS of 2 [48]. Coprimary endpoints were OS and safety in the per-protocol population. Amongst 47 patients who received durvalumab, median OS was 6.0 months (95% CI 4.0–10.0), with a 12-month survival rate of 31%. Treatment-related adverse events (AEs) of grade 3+ occurred in nine patients (19%). Although these OS data did not meet the authors’ pre-defined threshold of ‘clinically meaningful’—based on historical standards for platinum-doublet chemotherapy—they suggested a potential benefit in the subgroup of patients with a PD-L1 tumor proportion score ≥ 1 who had mOS of 11.0 months (PD-L1 1–49% mOS 11.0 months [95% CI 4.0–16.0], PD-L1 ≥ 50% 11.0 months [95% CI 0–NR]). Finally, the phase 3b CheckMate 817 trial by Ready et al. [49] assessed flat-dose nivolumab with weight-based ipilimumab in patients with advanced NSCLC. They recently reported on first-line treatment outcomes in patients with good ECOG PS (Cohort A) and special populations (Cohort A1), including those with ECOG PS of 2, untreated BMs and chronic liver/renal disease. Amongst 139 patients in Cohort A1 with ECOG PS of 2, mOS was 9.0 months (95% CI 5.5–12.9), with a 3-year OS rate of 18.7%. Although not designed to compare cohorts, it is worth noting that mOS in Cohort A was 16.8 months (95% CI 14.6–22.4), with a 3-year OS rate of 33.7%. Grade 3+ treatment-related AEs were similar between cohorts, both ~30%. Although limited in interpretation due to trial design, these data would not necessarily give clinicians confidence in choosing ICIs over alternative systemic therapy for patients with poor ECOG PS.

The recently published IPSOS trial is the first published randomized clinical trial that specifically sought to compare the efficacy of ICIs against single-agent chemotherapy in patients who were ineligible for platinum-doublet chemotherapy—either on the basis of ECOG PS 2–3, or >70 years old with significant comorbidities [5]. The trial enrolled 453 patients and was randomized in a 2:1 ratio to atezolizumab or single-agent chemotherapy (vinorelbine or gemcitabine). The primary outcome was OS, assessed in the intention-to-treat population. Safety and patient-reported health-related quality of life were among other key secondary endpoints. Among those enrolled, the median age was 75 (IQR 69.0–80.0), and 83% had ECOG PS of 2–3 (n = 344 ECOG PS 2, n = 34 ECOG PS 3). With a median follow-up of 41.0 months (IQR 36.7–47.8), atezolizumab led to a significantly longer mOS than chemotherapy (10.3 months versus 9.2 months, HR 0.78; 95% CI 0.63–0.97). There was a doubling of 24-month survival, with 24% in the atezolizumab group versus 12% in the chemotherapy group. The authors noted clinically meaningful improvement (for appetite, constipation, dyspnea, cough, and chest pain) or stability (all others) for symptoms in the atezolizumab group, with clinically meaningful deteriorations across several domains in those receiving chemotherapy. In safety analyses, grade 3+ treatment-related AEs were reported in 16% of patients receiving atezolizumab, versus 33% of patients receiving chemotherapy. Although the 1-month improvement in mOS is certainly modest, the doubling of two-year survival rates and the improved safety profile relative to single-agent chemotherapy are encouraging. It remains to be seen how these findings are integrated into consensus guidelines, and ultimately clinical practice.

When considered altogether, the available data suggest that the benefit of ICIs in patients with poor ECOG PS is modest, especially when compared to patients with good ECOG PS, as seen in the majority of landmark trials. Based on older data highlighting safety concerns around utilizing platinum-doublet chemotherapy in this patient population [50], single-agent ICIs may very well become the standard of care based on the results from IPSOS. However, these data have not yet been incorporated into consensus guidelines. Notably, in the most recent clinical practice guidelines from the European Society of Medical Oncology (ESMO) [51], ICI monotherapy was recommended for first-line treatment in patients with ECOG PS of 2 and PD-L1 ≥ 50%, but in patients with ECOG PS of 2 and PD-L1 < 50%, cytotoxic chemotherapy alone was recommended over combination ICI–chemotherapy strategies based on the lack of data in this population.

In terms of other ICI-containing strategies for patients with poor ECOG PS, it is worth highlighting the eNERGY-GFPC 06-2015 study [52]. This phase 3 trial randomized patients with poor ECOG PS (or older age) to nivolumab and ipilimumab or platinum doublet chemotherapy. The data monitoring committee halted recruitment of patients with poor ECOG PS because of high mortality (mOS 2.9 months versus 6.1 months, HR 1.8; 95% CI 0.99–3.3 for nivolumab and ipilimumab versus platinum-doublet chemotherapy, respectively). At the present time, there are no prospective data evaluating the combination of ICIs with single-agent chemotherapy in patients with poor ECOG PS, but this could be an important area for future study.

### 2.2. Patients with Autoimmune Disease (AID)

It has become well-established that ICIs are associated with the potential for development of unique AEs compared with cytotoxic chemotherapy through off-target impacts of immune system activation. The incidence of immune-related AEs (irAEs) from ICIs in the NSCLC literature varies, but any-grade IrAEs occur in ~30% of treated patients and grade 3+ irAEs occur in ~15% of patients. Nearly every landmark phase 3 trial of ICIs has excluded patients with a history of AID who have required systemic therapy due to fears of either the increased incidence of de novo IrAEs or flares of the underlying AID. Exceptions to these exclusion criteria generally include those with autoimmune hypothyroidism stable on physiologic replacement, and those with autoimmune-mediated cutaneous conditions without the need for systemic immunosuppression (Table 1 and Table 2). As it is estimated that 14–25% of NSCLC patients have a concomitant AID [53], incomplete understanding of the safety and efficacy of ICIs in this patient population represents a crucial knowledge gap.

As outlined in a recent review paper by Tison et al. [54], prospective data on the use of ICIs in patients with AID are lacking, especially in those with NSCLC. In the limited non-randomized prospective data available [55,56,57,58], flares of pre-existing AID occurred in 11–24% of patients, with any irAE occurring in 44–50%. In two retrospective studies from Canadian groups containing sizeable proportions of patients with NSCLC, flares of baseline AID occurred in 15–30% of patients, and there were no associations between baseline AID and the development of irAEs or adverse survival outcomes [59,60].

The most robust data on the use of ICIs in patients with NSCLC and known AID come from a systematic review and meta-analyses by Aung et al. [61]. They extracted data from 24 cohort studies, including 11,567 patients with cancer (3774 with NSCLC) and 1157 with AID (250 in those with NSCLC). In those with NSCLC, flares of known AID occurred in 21% of cases. The incidence of any new irAE was 40% in those with AID and 34% in those without, representing a significantly higher risk (RR 1.51; 95% CI 1.12–2.03). This relationship was consistent when analyzing grade 3+ IrAEs (RR 1.95; 95% CI 1.01–3.75). Interestingly, patients with NSCLC and AID were significantly more likely to have an objective disease response to ICIs (24% versus 19%, RR 1.56; 95% CI 1.19–2.04). This latter point is consistent with several published studies indicating an association between the development of irAEs and improved clinical outcomes in patients with NSCLC [62,63,64]. The primary limitation of this study is the inability to ascertain the baseline AID activity, and how patients were being managed prior to the initiation of ICIs. In totality, although there may be a modestly increased relative risk of AID flare and the onset of irAEs, the absolute risk is low, and these safety considerations should be made in the context of potential association with an enhanced anti-tumor immune response.

Importantly, neither the American Society of Clinical Oncology (ASCO) nor ESMO definitively recommend against the use of ICIs in patients with baseline AID in their most recent guidelines [65,66]. Rather, both consensus guidelines provide general suggestions for multidisciplinary discussion, the assessment of baseline AID stability and the replacement of non-specific systemic immunosuppressive agents with targeted agents where possible. Ultimately, randomized data in this space may be difficult to acquire given the heterogeneity of AID and the diverse group of malignancies for which ICIs are currently used.

### 2.3. Older Patients

It is estimated that >35% of new NSCLC diagnoses are made in individuals ≥75 years of age, but the majority of clinical trials that have established the efficacy of ICIs in non-oncogene-driven NSCLC enrolled patients with a median age of ~65. Although age is not in itself an exclusion criteria, the increased incidence of co-morbidities and diminished ECOG PS amongst older adults are both likely to contribute to the under-representation of this patient population. Furthermore, there is a theoretical biological underpinning to the potential for reduced efficacy of ICIs in older adults due to age-related immune senescence—a topic reviewed comprehensively by Elias et al. [67].

Several retrospective cohort studies have analyzed the outcomes of older patients with NSCLC treated with ICIs, with concordant findings amongst them [29,68,69,70]. For example, Youn et al. [68] analyzed the outcomes of 1256 patients ≥65 years old with NSCLC treated with single-agent ICI. mOS for the entire cohort from the time of treatment initiation was 9.3 months (95% CI 8.5–10.5), with age not being an independent prognostic factor for poor survival. Similarly, Grosjean et al. reported on 327 consecutive patients treated with first line pembrolizumab for patients with NSCLC and PD-L1 ≥ 50% [29]. In their analysis, 52% of patients were ≥70 years of age at treatment initiation. mOS for the entire cohort was 11.2 months (95% CI 8.8–15.3), with no survival differences between those ≥70 and <70 years of age. Moreover, there were no differences between the incidence of clinically significant irAEs or irAE-related hospitalizations between groups. More recently, Tsukita et al. [70] published data on 1245 consecutive patients with advanced NSCLC from 58 centers in Japan, all of whom were ≥75 years of age. In their cohort, the median age was 78 (range 75–95); 28% were treated with ICI plus chemotherapy, 34% were treated with an ICI alone, 25% with platinum-doublet chemotherapy and 12% with single-agent chemotherapy. mOS improved in those that received ICI plus chemotherapy (mOS 20.0 months [95% CI 17.1–23.6]) and ICI monotherapy (19.8 months [95% CI 16.5–23.8]) compared to those that received platinum-doublet chemotherapy (12.5 months [95% CI 10.7–15.6]) or single-agent chemotherapy (9.5 months [95% CI 7.4–13.4]). After propensity score matching based on relevant clinicopathologic variables, there was no difference in mOS between ICI plus chemotherapy and ICI monotherapy in patients with PD-L1 ≥1%, although there was a significantly higher incidence of grade 3+ irAEs in the combination arm (24.3% versus 17.9%, *p* = 0.03). Thus, the authors suggest that ICI monotherapy is preferred to ICI plus chemotherapy in older adults with PD-L1-positive NSCLC.

In terms of prospective data, Felip et al. reported on the phase 2 CheckMate 171 trial in which patients with previously treated advanced squamous NSCLC received nivolumab [46]. Of the 811 patients, 278 (34%) were ≥70 years old and 125 (15%) were ≥75 years old. mOS for the entire cohort was 10.0 months (95% CI 9.2–11.2), which was comparable to those patients ≥70 (10.0 months [95% CI 8.3–11.4]) and ≥75 (11.2 months [95% CI 7.9–14.2]). Furthermore, both subgroups of older patients had a similar occurrence of grade 3+ treatment-related AEs compared to the entire cohort (16% versus 18% versus 14%). The aforementioned phase 3b/4 CheckMate 153 trial included patients with previously treated NSCLC receiving nivolumab in the second line or later. Among 1426 patients included, 556 (39%) were ≥70 years old. mOS was 9.1 months (95% CI 8.3–10.4) in the entire cohort, which was comparable to patients ≥70 years old (10.3 months [95% CI 8.3–11.5]). [31] Again, the incidence of grade 3+ treatment-related AEs was similar. The ELDERS study was a prospective observational study that sought to investigate the impact of age on ICI-related toxicity in patients with NSCLC and melanoma [71]. The authors also evaluated the potential role of pre-screening elderly patients for frailty with the Geriatric-8 (G8) tool. Of the 140 included, 50% were ≥70 years old. Importantly, they did not find a significant difference in the incidence of grade 3+ irAEs between the younger and older cohort (12.9% versus 18.6%, OR 1.55; 95% CI 0.61–3.89). Although they found that a positive G8 screening was a predictor of admission to hospital, the majority of admissions were not as a result of ICI toxicity. The authors concluded that the G8 tool may be of value if routinely included in pre-ICI assessments with the intention to offer holistic geriatric assessments if positive.

An additional study to consider is the systematic review and meta-analysis by Kim et al. [72]. They included phase 2/3 clinical trials in which patients were randomized to ICIs versus the standard of care across several tumor sites. In total, 30 studies comprising 17,476 patients were included, of which 10 studies were in NSCLC, representing 6009 patients (48% ≥ 65 years old). In this subgroup analysis, they did not find any difference in the HR for mOS (HR 0.71; 95% CI 0.61–0.84 versus HR 0.78; 95% CI 0.68–0.90) nor progression-free survival (PFS) (HR 0.75; 95% CI 0.52–1.08 versus HR 0.85; 95% CI 0.64–1.12) when comparing patients ≥65 years old with those <65 years old.

There are two prospective randomized trials which were specifically designed to include a higher proportion of older adults, eNERGY-GFPC 06-2015 and IPSOS—both of which have been reviewed above in the context of patients with poor ECOG PS. The eNERGY-GFPC 06–2015 study, which randomized participants to nivolumab–ipilimumab or platinum-doublet chemotherapy, included patients ≥70 years old with favorable ECOG PS. Of the 204 patients included in the updated analysis, the median age was 74 years. In the subgroup analysis of older patients, there was a significant improvement in mOS with nivolumab–ipilimumab compared to platinum-doublet chemotherapy, 22.6 months (95% CI 18.1–36.0) versus 11.8 months (95% CI 8.9–20.5) [52]. The IPSOS trial compared atezolizumab to single-agent chemotherapy in patients classically seen to be unfit for platinum-containing chemotherapy [5]. Of 453 randomized patients, ~70% were ≥70 years old, and ~30% were ≥80 years old. Although the interpretation of subgroup analyses must be undertaken with caution, the benefit of atezolizumab was retained in patients aged 70–79 years old (HR 0.68, 95% CI 0.49–0.94), but not in patients ≥80 years old (HR 0.97, 95% CI 0.66–1.44).

Taken together, the available data do not suggest that older age meaningfully impacts clinical outcomes or safety for patients with NSCLC treated with ICIs. It remains to be seen whether comprehensive geriatric assessments prior to ICIs have a beneficial impact on patients’ fitness and quality of life and help to evaluate the risk of treatment toxicity as they do for older patients treated with cytotoxic chemotherapy [73].

### 2.4. Patients with Brain Metastases (BMs)

Approximately 25% of patients with NSCLC will develop BMs during the course of their illness. Although radiotherapy is the primary modality by which BMs are treated, its use can be associated with both acute and chronic toxicities, impacting patient function and quality of life [74,75]. Unlike with cytotoxic chemotherapy, patients treated with TKIs for oncogene-driven NSCLC can experience a significant intracranial response, which potentially abrogates the immediate need for radiotherapy [76,77,78,79]. The efficacy of ICIs for patients with active BMs is less clear, as every landmark phase 3 clinical trial evaluating the efficacy of ICIs in patients with advanced NSCLC has excluded patients with symptomatic and/or untreated BMs (Table 1 and Table 2). Although several retrospective cohort studies [80,81,82,83] and post-hoc exploratory analyses of phase 3 trials [84,85,86,87] have reported on the impact of ICIs on patients with NSCLC and BMs, prospective data evaluating those with active BMs are limited.

The first prospective study of ICIs in patients with active brain metastases was published by Goldberg et al. [88]. They conducted a single-center phase 2 trial of pembrolizumab in patients with stage IV NSCLC and at least one brain metastasis 5–20 mm in size, not previously treated or progressing after previous radiotherapy, with no neurologic symptoms or requirement of oral corticosteroids. Only patients whose ECOG PSs were <2 were included. Overall, 42 patients were treated, and analyzed in two cohorts: cohort 1 (n = 37) for those with PD-L1 expression of ≥1% and cohort 2 (n = 5) for those with PD-L1 <1%, or unevaluable. The primary endpoint was the proportion of patients achieving an intracranial response. Half of patients received local BM treatment prior to enrollment (stereotactic radiosurgery [n = 16], whole-brain radiotherapy [n = 8] and surgical resection [n = 4]). Median follow-up was 8.3 months (IQR 4.5–26.2). In cohort 1, 11 (30%) of patients had a documented intracranial response, with four complete responses (11%). Median duration of response was 5.7 months (IQR 4.0–17.7). An additional four patients (11%) had stable disease, whilst 16 patients (43%) had primary progression, and six (14%) were unevaluable. There were no responses observed in cohort 2. Among 27 patients who were evaluable for both CNS and systemic response, six had discordant outcomes—half of these patients had CNS progression with systemic response, and the other half had the opposite scenario. In a post-hoc exploratory analysis, CNS PFS was 2.3 months (95% CI 1.9-NR), with 33% of patients without CNS progression at 1 year. mOS was 9.9 months (95% CI 7.5–29.8), with estimated 1- and 2-year OS rates of 40% and 34%, respectively. The incidence of serious treatment-related AEs was 14%, in keeping with the known safety profile of pembrolizumab. Grade 3 neurologic AEs were reported in three patients (cognitive dysfunction, seizure or stroke)—none of which were deemed attributable to pembrolizumab. Limitations of this study include a single-arm design, the exclusion of patients with symptomatic BMs and the exclusion of patients requiring corticosteroids. Despite these limitations, the authors concluded that patients with NSCLC and untreated or progressing BMs may benefit from systemic therapy with pembrolizumab.

In a more recent single-arm phase 2 trial by Nadal et al. [89], patients with non-squamous NSCLC and untreated BMs (without neurologic symptoms) or those who were asymptomatic after medical management were administered atezolizumab, carboplatin and pemetrexed in the standard fashion. Notably, patients receiving up to 4 mg of oral dexamethasone per day were included. The primary endpoints were PFS at 12 weeks, and incidence of grade 3+ AEs during the first 9 weeks. 40 patients were enrolled, and 22 (55%) received corticosteroids at baseline. The 12-week PFS rate was 62%, with a 28% risk of a grade 3+ AE during the first 9 weeks. With a median follow-up of 31 months, intracranial response rate was 43%, with a systemic response rate of 45%. Of the 17 patients with intracranial response, five were complete responses. Median time to intracranial response was 82 days, with median duration of intracranial response of 14 months (95% CI 10.0–NR). In exploratory analyses, they found that both intracranial and systemic ORR were comparable irrespective of the concurrent use of oral corticosteroids. mOS was 11.8 months (95% CI 7.6–16.9), and the estimated 1- and 2-year OS rates were 50% and 28%, respectively. Finally, they reported that 24 (60%) of patients received radiotherapy to the brain as a result of intracranial progression—16 received whole-brain radiotherapy, and 8 received stereotactic radiosurgery. Median time to receipt of brain radiotherapy was 10.9 months (95% CI 7.8–15.9). From their results, the authors concluded that combination chemoimmunotherapy may allow for the safe delay of whole-brain radiotherapy in properly selected patients. Although data from both these studies are encouraging, randomized trials to determine the optimal treatment approach for this patient population are needed.

## 3. Molecular Characteristics

### 3.1. EGFR & ALK

*EGFR* mutations and *ALK* fusions are among the most common druggable oncogenic driving events in non-squamous NSCLC, occurring with incidences of ~15% [90] and ~5%, [91] respectively. Although the majority of patients with these oncogenic drivers have durable responses to treatment with their respective first-line TKIs, resistance inevitably develops over time. Until recently, the majority of data examining the outcomes of ICIs in patients with *EGFR* mutations or *ALK* translocations have been retrospective, or from small subgroups of clinical trials such as CheckMate 057 [92], IMpower 150 [93] and OAK [18]. Qiao et al. authored a comprehensive review of ICIs in EGFR-mutated NSCLC, but the clear conclusion of their review highlighted the limited efficacy of ICIs in these patients [90]. As such, our focus will be on summarizing the recent prospective randomized data in this space.

The first phase III trial to examine the efficacy of ICIs post-TKI progression in EGFR-mutated NSCLC was ORIENT-31 by Lu et al. [94]. This randomized, double-blind trial was conducted in 52 hospitals across China. Key inclusion criteria were age 18–75, ECOG PS 0–1, and disease progression after first-line TKI. The primary endpoint was PFS. The trial randomly assigned 476 patients in a 1:1:1 ratio to receive the PD-1 inhibitor sintilimab plus IBI305 (anti-VEGF) plus chemotherapy, sintilimab plus chemotherapy or chemotherapy alone. At the second interim analysis, median PFS with sintilimab plus chemotherapy was significantly longer than with chemotherapy alone (5.5 months [95% CI 4.5–6.1] versus 4.3 months [95% CI 4.1–5.3]; HR 0.72, 95% CI 0.55–0.94). Although not powered to formally test OS, the mOS in these groups were similar: 20.5 months (95% CI 15.8–25.3) versus 19.2 months (95%CI 15.8–22.4); HR 0.97, 95% CI 0.71–1.32. There was also no significant difference in time to deterioration in EORTC QLQ-C30 Global Health Status Dimension Score with the addition of sintilimab to chemotherapy. As this trial was only conducted in Chinese patients, the results are difficult to extrapolate to other patient populations. However, a 1.2-month PFS benefit without significant differences in OS is of questionable clinical significance.

There have been three other phase III trials evaluating the impact of ICIs as part of latter-line treatment in patients with oncogene-driven NSCLC. First, the CheckMate-722 trial randomized 294 patients from USA and southeastern Asia with *EGFR*-mutated NSCLC and progression on first- (or second-)line TKI 1:1 to receive nivolumab and chemotherapy or chemotherapy alone [38]. The primary endpoint was PFS. At final analysis (minimum 18.2 months) there was no significant improvement in PFS (5.6 months versus 5.4 months, HR 0.75; 95% CI 0.56–1.00) or OS (19.4 months versus 15.9 months, HR 0.82; 95% CI 0.61–1.10). Higher rates of grade 3–4 treatment-related AEs were seen with the addition of nivolumab (45%) versus chemotherapy alone (29%).

Next, the KEYNOTE-789 trial randomized 492 patients with *EGFR*-mutated NSCLC and progression after TKI treatment 1:1 to receive pembrolizumab plus chemotherapy or chemotherapy alone. Notably, there was a diverse patient population with enrollment from North America, South America, Australia, Europe and Asia. Co-primary endpoints were PFS and OS. At final analysis, there was no significant difference between the groups in terms of PFS (5.6 months versus 5.5 months, HR 0.80; 95% CI 0.65–0.97) or OS (15.9 months versus 14.7 months, HR 0.84; 95% CI 0.69–1.02). Slightly higher rates of grade 3–4 AEs were also seen with the addition of pembrolizumab (44%) versus chemotherapy alone (39%).

Finally, recent results from the phase III ATTLAS trial are the first to demonstrate the potential benefit of adding ICIs to the treatment approach of oncogene-driven NSCLC in latter-line settings [37]. The rationale for the ATTLAS trial came from the results of a subgroup analysis of the phase III IMpower 150 study, where the addition of atezolizumab to bevacizumab/carboplatin/paclitaxel (ABCP) led to numerically longer OS than with bevacizumab/carboplatin/paclitaxel (BCP) in patients with sensitizing *EGFR* mutations [93]. The ATTLAS trial enrolled 228 patients (215 *EGFR* mutation, 13 *ALK* translocation) from 16 sites in the Republic of Korea and randomized them in a 2:1 ratio to either ABCP or carboplatin (or cisplatin) plus pemetrexed (PC). The primary endpoint was investigator-assessed PFS, with key secondary endpoints of OS and ORR. Notably, no crossover to atezolizumab was permitted. Clinical endpoints were evaluated in a modified intention-to-treat analysis of patients, defined as those who received at least one study treatment. At a median follow-up of 26.1 months (IQR 24.7–28.2), median PFS was significantly longer with ABCP versus CP (8.5 months versus 5.6 months, HR 0.62; 95% CI 0.45–0.86, *p* = 0.004). The ORR was also significantly higher in ABCP than in PC (69.5% versus 41.9%, *p* < 0.01). Despite these findings, there was no difference in mOS (20.6 months versus 20.3 months, HR 1.01; 95% CI 0.69–1.46). Furthermore, grade 3+ AEs were more than twice as frequent in ABCP (35%) than in PC (15%), with more than half of patients (54%) in ABCP requiring treatment interruption or dose modification. In subgroup analyses, PFS was not significantly different with ABCP in patients with *EGFR* deletion 19 (HR 0.69; 95% CI 0.44–1.08), or those with acquired Thr790Met (HR 1.07; 95% CI 0.59–1.94). However, there was favorable PFS in those with EGFR Leu858Arg (HR 0.52; 95% CI 0.31–0.88). The small number of patients with *ALK* translocations makes drawing conclusions difficult.

There are several limitations to the ATTLAS trial. First, similar to ORIENT-31, the generalizability of this trial to broader patient populations is uncertain, given that enrollment in this trial came from a single country. Second, it is unclear why bevacizumab was omitted from the control group, especially when it was included in IMpower-150. Third, the majority of patients were pre-treated with first- or second-generation TKIs (54%), as opposed to third-generation TKIs which are the current standard of care. Overall, the lack of an OS benefit paired with a clinically significant increase in serious treatment-related AEs in ABCP versus CP lead to the conclusion that the overall clinical importance of this therapeutic strategy seems to be marginal.

The sum of all these trials to date indicate there is a limited role for the addition of ICIs to the latter-line treatment of oncogene-driven NSCLC, especially in those with *EGFR* mutations. There are limited data in those with *ALK* translocations. Future clinical trials should evaluate whether there is a defined role for the addition of ICIs in this space, such as in those patients with greater expression of PD-L1, or in specific *EGFR* mutations like Leu858Arg.

### 3.2. Other Molecular Alterations: ROS1, BRAF

*BRAF* mutations are detected in ~5% of patients with NSCLC, with similar distribution of V600E and non-V600E mutants [95]. The combination of the *BRAF* inhibitor dabrafenib and *MEK* inhibitor trametinib have FDA approval for treatment of *BRAF* V600E-mutated NSCLC based on the results of phase II data, with ORR of ~65% in both treatment-naïve and pre-treated patients [96]. At present, there are no prospective data evaluating the efficacy of ICIs in *BRAF*-mutated NSCLC. Several modestly sized retrospective studies (n = 15 to n = 46) of *BRAF*-mutated NSCLC patients treated with ICIs demonstrate ORR comparable to those seen in landmark clinical trials [97,98,99,100]. Although the small study sizes and retrospective nature limit any definitive conclusions, there does seem to be a potential trend toward improved ORR in patients with non-V600E *BRAF* mutations, potentially due to enrichment amongst patients with a significant smoking history and thus an increased tumor mutation burden. As summarized in a review paper by Tabbo et al. [95], there are pre-clinical data to suggest that BRAF and MEK inhibitors may induce a favorable immune microenvironment, thus rationalizing a combination treatment strategy including ICIs. The B-FAST trial (NCT 03178552) is an ongoing phase II/III study enrolling patients with advanced NSCLC harboring actionable somatic mutations in the blood, with cohort E being patients with BRAF V600E mutations and treated with vemurafenib, cobimetinib and atezolizumab.

ROS1 fusions are oncogenic drivers in ~1% of non-squamous NSCLC, and first-line treatment is with TKIs such as entrectinib or crizotinib [101,102]. There are no prospective data evaluating the use of ICIs in patients with ROS1 fusions, and the retrospective data are also limited. In a study by Mazieres et al., one out of six patients with a ROS1 fusion had an objective response when treated with single-agent ICIs [99]. Another retrospective study from Choudhury et al. found a similarly low ORR in this patient population, with two of 16 patients with ROS1 fusions having a response to single-agent ICIs [103]. In contrast, they reported an ORR of 83% (n = 6) for those receiving both chemotherapy and ICIs. There were no differences in PD-L1 expression or tumor mutational burden between responders and non-responders. These results are in keeping with those reported by Huang et al., who observed an ORR of 83% (n = 6) for the combination of ICIs and chemotherapy in the frontline setting for patients with *ROS1* fusions [104]. Notably, the baseline ORR from chemotherapy alone in *ROS1*-fusion NSCLC is 53% (n = 48) [105]. Given these are retrospective studies of limited size, no conclusions can be made about the additional benefit of ICIs compared to chemotherapy alone in patients with *ROS1* fusions, and ICIs with chemotherapy certainly do not supplant TKIs in the first-line treatment setting.

## 4. Conclusions

Since the initial FDA approval of an ICI for the treatment of non-oncogene-driven NSCLC 9 years ago, this therapeutic strategy has been cemented as a crucial component of treatment for the majority of these patients. However, there is a clear efficacy–effectiveness gap whereby patients in the ‘real world’ seem to have more modest clinical outcomes compared to those enrolled in landmark clinical trials. This may be driven by the under-representation of important patient populations, including populations defined by clinical or molecular characteristics. In this review, we summarized the data outlining the evidence of ICIs in patients with poor ECOG PS, underlying AID, older age, active BMs, and molecular aberrations such as *EGFR* mutations, *ALK* fusions, *BRAF* mutations and *ROS1* fusions. Although ICIs may have a role in the treatment paradigm for some of these subgroups, consistent high-quality randomized data are lacking. It is our hope that these patients are included in landmark clinical trials where the available evidence supports their inclusion (underlying AID, older patients), or separate trials are designed around physiologically vulnerable patients who constitute a substantial proportion of real-world patients (poor ECOG PS, active BMs). Although ICIs do not seem to have clearly meaningful clinical activity in those with *EGFR* mutations, *ALK* fusions or *ROS1* fusions, there are ongoing studies to determine if those with *BRAF* mutations may benefit.

## Figures and Tables

**Table 1:** Characteristics of landmark clinical trials of immune checkpoint inhibitors for Stage IV NSCLC in the first-line setting.

Immune Checkpoint Inhibitor	Study (Year)	Treatment Arm	mOS Gain, HR (CI)	Treatment-Related Gr 3–5 AE, % (n)	Median Age (Range)	ECOG PS Enrolled	Autoimmune Disease	Brain Metastases	Molecular Alterations
Atezolizumab	IMpower 150 [1] (2018)	Atezolizumab + carboplatin + paclitaxel + bevacizumab	4.8, 0.80 (0.67–0.95)	57% (225)	63 (31–89)	0–1	Excluded ^1^	Treated, asymptomatic	Initially included, n = 35 (9%) EGFR+, n = 13 (3%) with ALK translocation ^2^
	IMpower130 [2] (2019)	Atezolizumab+ Carboplatin+ Nab-paclitaxcel	4.7, 0.79 (0.64–0.98)	75% (354)	64 (18–86)	0–1	Excluded ^1^	Treated, asymptomatic	Initially included, n = 32 (7%) with EGFR or ALK translocation ^2^
	IMpower110 [3] (2020)	Atezolizumab	7.1, 0.59 (0.40–0.89) ^3^	13% (37)	64 (30–81)	0–1	Excluded ^1^	Treated, asymptomatic	Initially included, n = 8 (3%) with EGFR or ALK translocation ^2^
	IMpower131 [23] (2020)	Atezolizumab+ Carboplatin+ Nab-paclitaxcel	N.S.	69% (232)	65 (23–86)	0–1	Excluded	Treated, asymptomatic	Included, n = 1 (<0.1%) EGFR+
	IMpower132 [4] (2021)	Atezolizumab + carboplatin/cisplatin + pemetrexed	N.S.	58% (170)	64 (31–85)	0–1	Excluded	Treated, asymptomatic	Excluded
	IPSOS [5] (2023)	Atezolizumab	1.1, 0.78 (0.63–0.97)	17% (52)	75 (N/A)	0–3	Excluded ^4^	Treated, asymptomatic	Non-exon 19 or Leu858Arg EGFR eligible, n = 1 (<1%).
Cemiplimab	EMPOWER-Lung-01 [6] (2021)	Cemiplimab	12.8, 0.57 (0.46–0.71) ^5^	18% (65)	63 (N/A)	0–1	Excluded ^6^	Treated, asymptomatic	Excluded
	EMPOWER-Lung-03 [7] (2022)	Cemiplimab + platinum-based chemotherapy	8.9, 0.71 (0.53–0.93)	44% (136)	63 (N/A)	0–1	Excluded	Treated, asymptomatic	Excluded
Durvalumab	MYSTIC [8] (2020)	Durvalumab + Tremelimumab	N.S.	23% (85)	65 (32–87)	0–1	Excluded ^7^	Treated, asymptomatic	Excluded
	POSEIDON [9] (2023)	Durvalumab + Tremelimumab + Chemotherapy	2.7, 0.77 (0.65–0.92)	55% (182)	63 (27–87)	0–1	Excluded ^8^	Treated, asymptomatic	Excluded
Nivolumab	CheckMate 026 [10] (2017)	Nivolumab	N.S.	18% (49)	63 (32–89)	0–1	Excluded	Treated, asymptomatic	Excluded
	CheckMate227 [11] (2019)	Nivolumab + Ipilimumab	2.2, 0.77 (0.66–0.91) ^9^	20% (81)	64 (26–87)	0–1	Excluded ^10^	Treated, asymptomatic	Excluded
	CheckMate-9LA [12] (2021)	Nivolumab+ Ipilimumab +Chemotherapy	4.8, 0.74 (0.62–0.87)	50% (180)	65 (N/A)	0–1	Excluded ^10^	Treated, asymptomatic	Excluded
Pembrolizumab	KEYNOTE-024 [13] (2016)	Pembrolizumab	12.9, 0.62 (0.48–0.81)	31% (48)	65 (33–90)	0–1	Excluded ^11^	Treated, asymptomatic	Excluded
	KEYNOTE-189 [15] (2018)	Pembrolizumab + platinum based chemotherapy	11.4, 0.60 (0.50–0.72)	67% (272)	65 (34–84)	0–1	Excluded ^12^	Treated, asymptomatic	Excluded
	KEYNOTE-407 [16] (2018)	Pembrolizumab + platinum based chemotherapy	5.6, 0.71 (0.59–0.85)	70% (194)	65 (29–87)	0–1	Excluded ^12^	Treated, asymptomatic	N/A
	KEYNOTE-042 [14] (2019)	Pembrolizumab	4.1, 0.79 (0.70–0.89)	18% (113)	63 (N/A)	0–1	Excluded ^12^	Treated, asymptomatic	Excluded
	KEYNOTE-598 [17] (2021)	Pembrolizumab + Ipilimumab	N.S.	62% (177)	64 (35–85)	0–1	Excluded ^12^	Treated, asymptomatic	Excluded

^1^ Patients with autoimmune hypothyroidism on stable doses of replacement and patients with type 1 diabetes on stable doses of insulin were eligible. ^2^ Patients with EGFR mutations or ALK translocations were ultimately excluded from the primary analyses during protocol amendment. ^3^ In an updated exploratory analysis of OS in the high PD-L1 expression group, mOS gain was 5.5 months, HR 0.76 (0.54–1.09). ^4^ Patients with autoimmune hypothyroidism on a stable dose of thyroid replacement were eligible. ^5^ mOS in the pre-specified PD-L1 ≥ 50% subgroup. ^6^ Patients with Type 1 diabetes on stable doses of insulin, vitiligo and hypothyroidism on stable doses of thyroid replacement were eligible. ^7^ Patients with vitiligo, alopecia, psoriasis not requiring systemic therapy or autoimmune hypothyroidism on stable doses of hormone replacement were eligible. ^8^ Patients with vitiligo, alopecia, autoimmune hypothyroidism on stable doses of hormone replacement and celiac disease were eligible. ^9^ mOS in the pre-specified PD-L1 ≥ 1% subgroup. ^10^ Patients with hypothyroidism on stable doses of hormone replacement, Type I diabetes and skin disorders not requiring systemic therapy were eligible. ^11^ Patients on physiologic hormone replacement for autoimmune disease were eligible in the absence of other systemic treatment, except patients with autoimmune hypothyroidism, who were excluded. If ≥2 years since last receipt of systemic treatment for autoimmune disease then patients were considered eligible. ^12^ Patients on physiologic hormone replacement for autoimmune disease were eligible in the absence of other systemic treatment. If ≥2 years since last receipt of systemic treatment for autoimmune disease then patients were considered eligible. N.S. = no significant difference. N/A = not applicable.

**Table 2:** Characteristics of landmark clinical trials of immune checkpoint inhibitors for Stage IV NSCLC in the 2+ line setting.

Immune Checkpoint Inhibitor	Study (Year)	Treatment Arm	mOS Gain, HR (CI)	Treatment-Related Gr 3–5 AE, % (n)	Median Age (Range)	ECOG PS Enrolled	Autoimmune Disease	Brain Metastases	Molecular Alterations
Atezolizumab	OAK [18] (2017)	Atezolizumab	3.5, 0.78 (0.69–0.89)	15% (90)	63 (33–82)	0–1	Excluded ^1^	Treated, asymptomatic	Included, n = 42 (10%) EGFR+, n = 2 (<1%) ALK translocation
Avelumab	JAVELIN Lung 200 [19] (2018)	Avelumab	N.S.	10% (39)	64 (N/A)	0–1	Excluded ^2^	Treated, asymptomatic	Included, n = 11 (3%) EGFR+, n = 1 (<1%) ALK translocation
Nivolumab	CheckMate 017 [20] (2015)	Nivolumab	3.2, 0.62 (0.48–0.79)	7% (9)	62 (39–85)	0–1	Excluded ^3^	Treated, asymptomatic	N/A
	Checkmate 057 [21] (2015)	Nivolumab	2.7, 0.70 (0.58–0.83)	10% (30)	61 (37–84)	0–1	Excluded ^3^	Treated, asymptomatic	Included, n = 44 (15%), EGFR+, n = 13 (4%) ALK translocation
Pembrolizumab	KEYNOTE-010 [22] (2016)	Pembrolizumab	3.4, 0.70 (0.61–0.80)	16% (110)	63 (N/A)	0–1	Excluded ^4^	Treated, asymptomatic	Included, n = 60 (9%) EGFR+, n = 6 (<1%) ALK translocation

^1^ Patients with stable autoimmune hypothyroidism on physiologic replacement and type 1 diabetes on a stable insulin regimen were eligible. ^2^ Patients with Type 1 diabetes, vitiligo, psoriasis or autoimmune thyroid disease not requiring immunosuppression were eligible. Patients requiring hormone replacement with corticosteroids were eligible if doses were less than or equivalent to 10 mg of prednisone per day. ^3^ Patients with vitiligo, type 1 diabetes, or autoimmune hypothyroidism on stable doses of replacement were eligible. ^4^ Patients with vitiligo were eligible. N.S. = no significant difference. N/A = not applicable.

## Data Availability

All data relevant to this manuscript is included within the body.
